# Histone deacetylases play distinct roles in telomeric *VSG* expression site silencing in African trypanosomes

**DOI:** 10.1111/j.1365-2958.2010.07284.x

**Published:** 2010-07-22

**Authors:** Qiao-Ping Wang, Taemi Kawahara, David Horn

**Affiliations:** 1 London School of Hygiene and Tropical MedicineKeppel Street, London WC1E 7HT, UK; 2 Center for Parasitic Organisms, State Key Laboratory of Biocontrol, School of Life Sciences, Key Laboratory of Tropical Diseases Control, Ministry of Education, Zhongshan Medical College, Sun Yat-Sen UniversityGuangzhou 510275, China

## Abstract

African trypanosomes evade the host immune response through antigenic variation, which is achieved by periodically expressing different variant surface glycoproteins (VSGs). *VSG* expression is monoallelic such that only one of approximately 15 telomeric *VSG* expression sites (ESs) is transcribed at a time. Epigenetic regulation is involved in *VSG* control but our understanding of the mechanisms involved remains incomplete. Histone deacetylases are potential drug targets for diseases caused by protozoan parasites. Here, using recombinant expression we show that the essential *Trypanosoma brucei* deacetylases, DAC1 (class I) and DAC3 (class II) display histone deacetylase activity. Both DAC1 and DAC3 are nuclear proteins in the bloodstream stage parasite, while only DAC3 remains concentrated in the nucleus in insect-stage cells. Consistent with developmentally regulated localization, DAC1 antagonizes SIR2rp1-dependent telomeric silencing only in the bloodstream form, indicating a conserved role in the control of silent chromatin domains. In contrast, DAC3 is specifically required for silencing at *VSG* ES promoters in both bloodstream and insect-stage cells. We conclude that DAC1 and DAC3 play distinct roles in subtelomeric gene silencing and that DAC3 represents the first readily druggable target linked to *VSG* ES control in the African trypanosome.

## Introduction

*Trypanosoma brucei* is a mono-flagellated parasitic protozoan that causes sleeping sickness in humans and Nagana in cattle. *T. brucei* branched very early from the eukaryotic lineage and displays some unusual features such as polycistronic transcription of protein-coding genes (Vanhamme *et al*., 2001). The *T. brucei* bloodstream-form cell surface comprises a dense variant surface glycoprotein (VSG) coat, encoded in a polycistronically transcribed telomeric *VSG* expression site (ES), which can periodically change to evade host immune defences (Pays, 2005). Although there are approximately 15 different telomeric *VSG* ESs, only one at a time is transcriptionally active (Hertz-Fowler *et al*., 2008). The mechanism underlying this monoallelic *VSG* expression remains unknown, although the telomeric location and chromatin remodelling appear to be involved; a telomere-binding protein known as repressor/activator protein 1 and a member of the ISWI family of SWI2/SNF2-related chromatin-remodelling complexes (ISWI) are required for efficient *VSG* ES silencing (Hughes *et al*., 2007; Yang *et al*., 2009). In addition, a dispensable histone methyltransferase (DOT1B) has a lesser role in *VSG* ES silencing, and is required for rapid transcriptional switching among ESs (Figueiredo *et al*., 2008).

Histone post-translational modifications (PTMs), in the N-terminal tails of histones in particular, play essential roles in chromatin assembly, DNA replication, recombination and repair, and transcriptional regulation (Groth *et al*., 2007; Kouzarides, 2007). Although *T. brucei* histones are divergent compared with other eukaryotes, several residues are acetylated or methylated and the functions of these PTMs may be conserved (Horn, 2007; Figueiredo *et al*., 2009). These histone PTMs are dynamically regulated by pairs of histone-modifying enzymes (Kouzarides, 2007). For example, histone deacetylases (HDACs) can reverse acetylation mediated by histone acetyltransferases (Drummond *et al*., 2005). HDACs participate in various forms of transcription repression such as telomere position effect in the yeast, *Saccharomyces cerevisiae* (DeRubertis *et al*., 1996; Rundlett *et al*., 1996)*,* position-effect variegation in *Drosophila* (Rundlett *et al*., 1996) and X-chromosome inactivation in mammals (Jeppesen and Turner, 1993). HDACs are also involved in many cancers and have emerged as drug targets for chemotherapy (Drummond *et al*., 2005). A large number of HDAC inhibitors have been developed, and related compounds now show potential for the treatment of diseases caused by protozoan parasites (Bougdour *et al*., 2009).

The roles of the *T. brucei* class III, Sir2-related deacetylases have been explored and it has been demonstrated that SIR2rp1, the only nuclear protein of this class in *T. brucei*, is required for basal telomeric silencing, but not for *VSG* ES silencing (Alsford *et al*., 2007). There are also four putative zinc-dependent, class I–II HDACs in *T. brucei* (Ingram and Horn, 2002), including DAC1 and DAC3, which appear to be essential for growth in the bloodstream form. In this study, we characterize these HDACs and demonstrate distinct roles in telomeric and *VSG* ES silencing.

## Results

### DAC1 and DAC3 are nuclear proteins

**Fig. 1:**
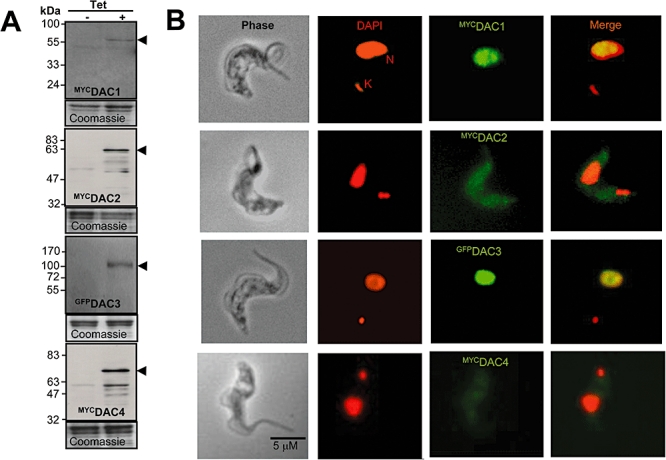
DAC1 and DAC3 are nuclear proteins. A. Western blot detection of tagged DACs 1–4 (arrowheads). The DACs were conditionally expressed from an ectopic locus in bloodstream-form cells. Cells were either cultured without Tet (−) or induced for 24 h with Tet (+, 1 µg ml^−1^). DACs 1–4 were predicted to be 43, 61, 75 and 64 kDa polypeptides respectively. DAC1 was fused to 12xMYC, while DAC2 and DAC4 were fused to a single MYC tag. DAC3 was fused to GFP. The Coomassie panels serve as loading controls. B. Immunofluorescence analysis. N, nucleus; K, kinetoplast (mitochondrial DNA). Other details as in A. GeneIDs: DAC1; Tb927.10.1680; DAC2, Tb11.01.7240; DAC3, Tb927.2.2190; DAC4, Tb927.5.2900.

To determine the sites of action of the various *T. brucei* DACs in trypanosomes, we initially established strains conditionally expressing N-terminally tagged versions of each protein (Fig. 1A). Interestingly, because we were particularly interested in the essential DAC1 and DAC3 as potential drug targets, ^MYC^DAC1 and ^GFP^DAC3 both accumulate in the nucleus, while ^MYC^DAC2 and ^MYC^DAC4 are distributed more widely and appear to be predominantly cytoplasmic (Fig. 1B); uninduced cells were consistently negative for GFP (data not shown). Further studies therefore focused on the essential, nuclear DACs. For the purpose of monitoring protein expression during RNA interference knockdown (see below), we integrated GFP tags at the *DAC1* and *DAC3* chromosomal loci in bloodstream-form cells. In this case, ^GFP^DAC1 and ^GFP^DAC3 were expressed under the control of native transcription. Once again, both proteins accumulated in the nucleus in bloodstream-form cells (Fig. S1 and see below).

### DAC1 and DAC3 display HDAC activity

**Fig. 2:**
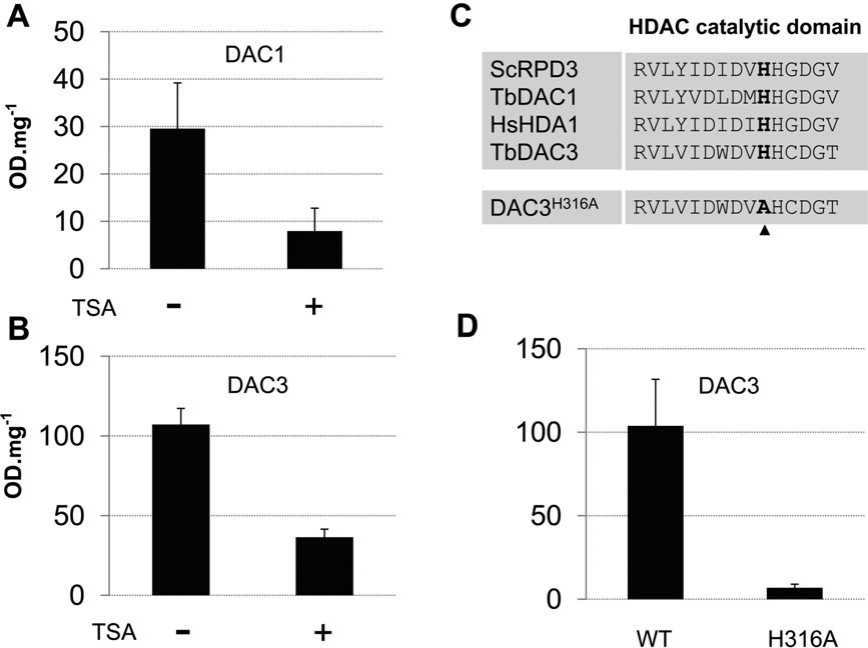
DAC1 and DAC3 display HDAC activity. A. DAC1 displayed HDAC activity that was subject to inhibition by Trichostatin A (TSA, 5 µM). Activity is expressed as OD_405_ per mg eluted protein. Assays were done in triplicate and error bars represent one sd. B. DAC3 displayed HDAC activity that was subject to inhibition by TSA (5 µM). Other details as in A. C. The conserved zinc-binding residue that forms part of the HDAC catalytic domain is indicated. Hs: *Homo sapiens*; Sc, *S. cerevisiae*; Tb, *T. brucei.* Accession numbers*:* ScRPD3, P32561; HsHDA1, P53973. D. The DAC3^H316A^ mutation abolishes HDAC activity. Other details as in A.

We demonstrated above that DAC1 and DAC3 are nuclear proteins in the bloodstream form. To determine whether these DACs display HDAC activity, we expressed recombinant DAC1 and DAC3 in *Escherichia coli*. Active, soluble DAC1 was obtained as an N-terminal MBP-fusion protein, whereas DAC3 was obtained as an N-terminal 6xHis-fusion protein. ^MBP^DAC1 and ^HIS^DAC3 were purified by affinity chromatography (Fig. S2) and HDAC activity was determined using an *in vitro* assay. Both proteins displayed HDAC activity (Fig. 2A and B), which may be relatively lower for DAC1 due to the presence of additional products in this preparation (Fig. S2A). Class I–II HDACs are zinc-dependent enzymes with conserved zinc-binding residues located at the base of the catalytic pocket; one His and two Asn residues participate in zinc-ion binding (Finnin *et al*., 1999). The purity and yield of the DAC3 preparation facilitated mutagenesis studies and by sequence alignment, residues Asn^314^, His^316^ and Asn^582^ in DAC3 were predicted to be these zinc ligands (Ingram and Horn, 2002). An H^316^A mutation (Figs 2C and S2C) abolished HDAC activity indicating that this residue is critical for the deacetylase activity of DAC3 (Fig. 2D).

### DAC1 antagonizes basal telomeric silencing

**Fig. 3:**
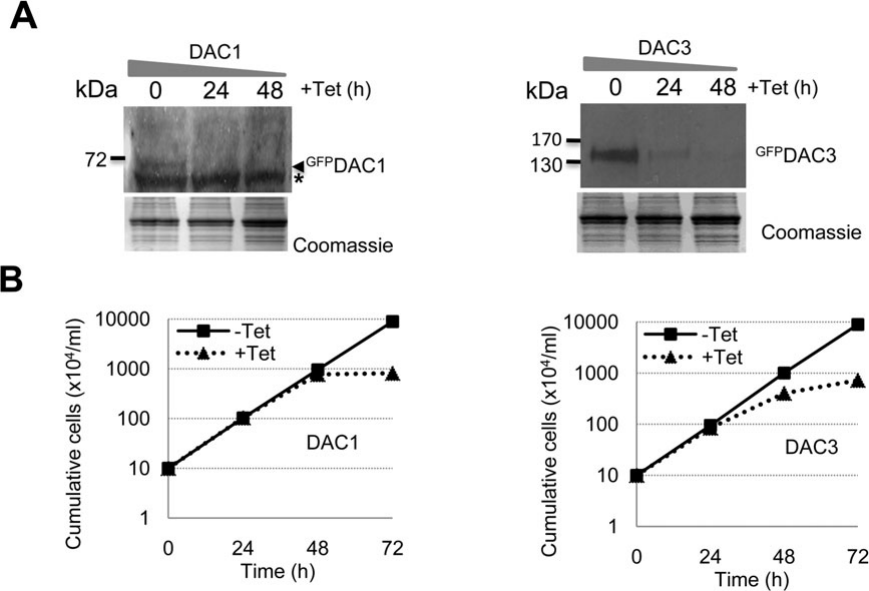
RNAi confirms that DAC1 and DAC3 are essential for growth in bloodstream-form trypanosomes. A. Expression of hairpin DAC1 or DAC3 cassettes triggers specific RNAi-mediated knockdown as reported by ^GFP^DAC1 and ^GFP^DAC3. The asterisk indicates a non-specific band and the Coomassie panels serve as loading controls. B. Growth curves for bloodstream-form cells during DAC1 or DAC3 knockdown (+ Tet). Four independent strains were analysed for each DAC. Error bars representing one sd are obscured by the data points.

In trypanosomes, many silent *VSG* genes are located at the telomeric end of polycistronic ESs. These transcription units have promoters that, unusually, recruit RNA polymerase I and are often located ∼50 kbp from the telomere. A small number of factors have been linked to the ES promoter silencing process (Hughes *et al*., 2007; Figueiredo *et al*., 2008; Yang *et al*., 2009), while distinct factors are required for basal telomeric silencing, which spreads only a few kbp from the telomere (Alsford *et al*., 2007; Kawahara *et al*., 2008). We used an RNA interference (RNAi) approach to assess the role of DAC1 and DAC3 in telomeric silencing. For this assay, hairpin RNAi knockdown constructs were engineered into a strain expressing ^GFP^DAC1 or ^GFP^DAC3 from one of the native alleles. Induction of RNAi against *DAC1* or *DAC3* leads to decreased ^GFP^DAC1 or ^GFP^DAC3 expression, respectively, indicating efficient protein knockdown in both cases (Fig. 3A). Each knockdown was associated with a growth defect (Fig. 3B) confirming that DAC1 and DAC3 are essential for growth in the bloodstream form.

**Fig. 4:**
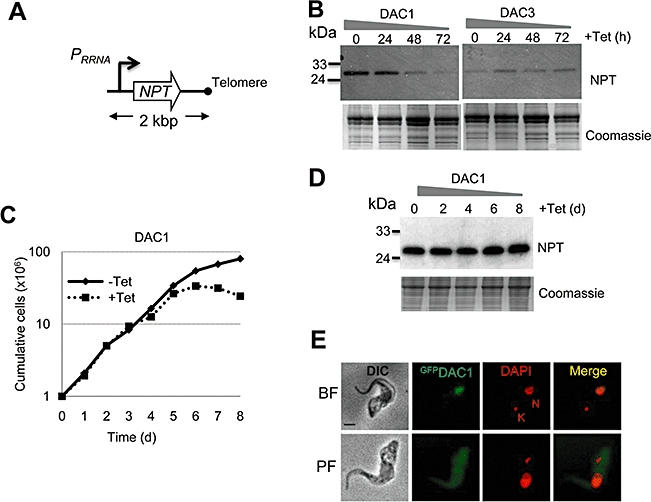
DAC1 antagonizes basal telomeric silencing in bloodstream-form trypanosomes. A. The schematic illustrates the *NPT* reporter under the control of an *rRNA* promoter located within 2 kbp of a telomere. B. Western blots showing *NPT* reporter expression in bloodstream-form cells during DAC knockdown (+ Tet). The Coomassie panels serve as loading controls. C. Growth curve for insect-stage cells during DAC1 knockdown (+ Tet). D. Western blots showing *NPT* reporter expression in insect-stage cells during DAC1 knockdown (+ Tet). The Coomassie panel serves as a loading control. The NPT signal here is stronger than seen in panel B because telomeric silencing is weaker in insect-stage cells (Glover and Horn, 2006). E. Localization of ^GFP^DAC1 expressed from the native locus in bloodstream form (BF) and procyclic form (PF) cells. N, nucleus; K, kinetoplast (mitochondrial DNA). Bar, 2 µm.

RNAi knockdown was then carried out in strains with an *NPT* reporter gene under the control of an *rRNA* promoter located within 2 kbp of a telomere (see Fig. 4A). Monitoring of *NPT* reporter expression revealed an increase in telomeric silencing specifically associated with DAC1 knockdown (Fig. 4B) and this finding was confirmed in an independent strain (data not shown) indicating that DAC1 antagonizes basal telomeric silencing. DAC1 knockdown also coincided with a growth defect in the insect stage (Fig. 4C) but knockdown had no impact on telomeric silencing in this life-cycle stage (Fig. 4D). This developmental stage-specific function for DAC1 is consistent with failure to accumulate in the nucleus in the insect, procyclic form (Fig. 4E); while ^GFP^DAC3 subcellular localization was not substantially altered following differentiation (Fig. S1), ^GFP^DAC1 displayed a strikingly different localization and appeared to be predominantly cytoplasmic. Cell cycle analysis also suggested a defect in DNA replication associated with DAC1 knockdown in bloodstream-form cells (Fig. S3); these cells apparently progress through mitochondrial DNA division without DNA replication.

### DAC3 is required for VSG ES silencing

**Fig. 5:**
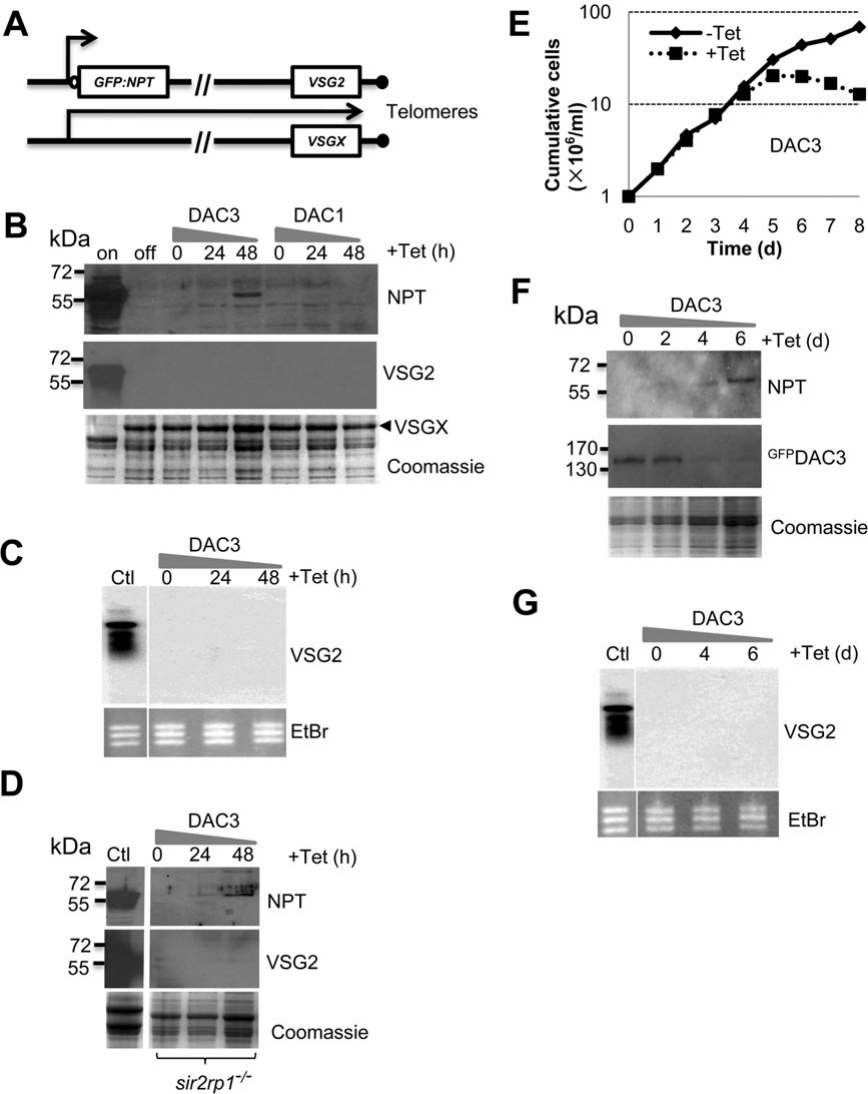
DAC3 controls *VSG* ES silencing. A. The schematic illustrates the silent *VSG2* ES with a Tet operator and *GFP:NPT* reporter adjacent to a silenced promoter. These bloodstream-form cells express a second *VSG (X)*. B. Western blots showing reporter expression in bloodstream-form cells during DAC knockdown (+ Tet). The ‘on’ sample with an active *VSG2* ES serves as a positive control and the ‘off’ sample serves as a negative control indicating that the *VSG2* ES remains repressed in the presence of Tet. The Coomassie panel serves as a loading control. C. Northern blot for *VSG2* expression in bloodstream-form cells during DAC3 knockdown. The ethidium bromide (EtBr) panel serves as a loading control. D. Western blotting showing reporter expression in *sir2rp1* null bloodstream-form cells during DAC3 knockdown (+ Tet). E. Growth curve for insect-stage cells during DAC3 knockdown (+ Tet). F. Western blots showing *NPT* expression during DAC3 knockdown in insect-stage cells. The Coomassie panel serves as a loading control. G. Northern blot for *VSG2* expression in insect-stage cells during DAC3 knockdown. The EtBr panel serves as a loading control.

There are approximately 15 telomeric and polycistronic *VSG* ESs in *T. brucei.* In bloodstream-form cells, all but one are reversibly repressed and the frequency of switching is very low, typically < 10^−6^ per population doubling (Horn and Cross, 1997). To determine whether DAC1 or DAC3 participate in transcriptional repression at *VSG* ESs, we generated a strain of *T. brucei* cells with a GFP-tagged *NPT* reporter immediate downstream of a reversibly silenced *VSG* ES promoter (Fig. 5A). We started with cells expressing *VSG2*, a single copy *VSG* on chromosome 6a (Melville *et al*., 2000). A construct with a Tet operator and the *NPT* reporter was integrated downstream of an active *VSG2* ES promoter under transcription permissive conditions. Tet removal allowed the Tet repressor to bind Tet operator and block *VSG2* transcription such that only cells that switch to an alternative *VSG* ES survive (Glover *et al*., 2007); these cells retain the *NPT* reporter downstream of the silenced *VSG2* ES promoter (Fig. 5A). *VSG2* ES inactivation and the capacity for reactivation were confirmed by VSG2 Western blot and GFP-NPT fluorescence analysis (data not shown).

Subsequently, we integrated the inducible *DAC1* or *DAC3* hairpin RNAi knockdown constructs into these cells. For each experiment, two independent strains were analysed and results from one of each pair are shown (Fig. 5B). Expression of the NPT reporter was specifically detected following DAC3 knockdown (Fig. 5B) and this phenotype was confirmed by detection of the GFP-tag on the NPT reporter (data not shown). Next, we determined whether DAC3 influences the silencing of *VSG2* located approximately 60 kbp downstream of the *VSG* ES promoter (Fig. 5A). Neither *VSG2* protein (Fig. 5B) nor mRNA (Fig. 5C) was detected in the samples that exhibited derepression adjacent to the promoter. These results indicated that DAC3 depletion led only to derepression at the *VSG* ES promoter, and that transcription was subsequently attenuated. This phenotype is similar to that reported for TbISWI knockdown (Hughes *et al*., 2007) and is consistent with the idea that additional factors are involved in *VSG* ES silencing (Horn, 2009). TbSIR2rp1 mediates basal telomeric silencing, but the effect does not extend to the *VSG* ES promoter (Alsford *et al*., 2007). To explore the possibility that DAC3 and SIR2rp1 cooperate to silence *VSG* ESs, we disrupted both *SIR2rp1* alleles in the DAC3 RNAi strain. These *sir2rp1* knockouts were confirmed by PCR (data not shown). However, expression of the *NPT* reporter and *VSG2* (Fig. 4D) were similar after DAC3 knockdown in the *SIR2rp1* wild-type and *sir2rp1* null strains (compare Fig. 4B and D). Parallel knockdown of both *DAC3* and *DAC1* did not lead to derepression of the *NPT* reporter (data not shown), suggesting that the DAC1 defect dominates when both deacetylases are targeted. Thus, neither SIR2rp1 nor DAC1 appear to cooperate with DAC3 to silence *VSG* ESs.

To ask whether DAC3 knockdown allows derepression of the *VSG2* ES in insect-stage cells, we differentiated the bloodstream-form DAC3 RNAi strains. DAC3 knockdown in these cells, once again, lead to a growth defect indicating that DAC3 expression is also essential for growth in the insect stage (Fig. 5E). Similar to the situation in bloodstream-form cells, the *NPT* reporter was derepressed (Fig. 5F) but *VSG2* remained undetectable (Fig. 5G), indicating that attenuation of transcription occurs in both life-cycle stages.

## Discussion

Previous studies revealed DAC1 and DAC3 as two putative essential deacetylases in *T. brucei* (Ingram and Horn, 2002) that are potential targets for chemotherapy (Horn, 2008). In this study, we report characterization of these enzymes using *in vitro* HDAC activity assays and functional analysis in trypanosomes. Our results indicate that both DAC1 and DAC3 display HDAC activity. We further demonstrate that DAC1 antagonizes telomeric silencing in bloodstream-form cells, and that DAC3 is required for *VSG* ES silencing in both bloodstream and insect-stage cells.

Based on sequence similarity, cellular localization, catalytic domain and mechanism of action, HDACs are divided into four major classes (De Ruijter *et al*., 2003). DAC1 and DAC2 are class I, and DAC3 and DAC4 are class II (Ingram and Horn, 2002). Trypanosomes also express three class III, Sir2-related deacetylases that are dispensable for growth; only one of these is a nuclear protein (Alsford *et al*., 2007). In human cells, class I HDACs are typically retained in the nucleus while class II HDACs are often able to shuttle between nucleus and cytoplasm (De Ruijter *et al*., 2003). We show that recombinant DAC1 and DAC3 are predominantly nuclear proteins in bloodstream-form trypanosomes, while DAC2 and DAC4 are predominantly cytoplasmic. Interestingly, DAC1 displays stage-specific subcellular localization, relocalizing to the cytoplasm in insect-stage cells.

Histone deacetylases are components of complexes that play important roles in transcription repression (Yang and Seto, 2008) and these complexes likely participate in transcription regulation in trypanosomes (Figueiredo *et al*., 2009). For example, histone H4K10 acetylation, mediated by the histone acetyltransferase, HAT2 (Kawahara *et al*., 2008), marks the transcriptional start sites for RNA polymerase II (Siegel *et al*., 2009). In addition, the class III deacetylase, SIR2rp1 (Alsford *et al*., 2007) and the histone acetyltransferase, HAT1 (Kawahara *et al*., 2008) are required for basal telomeric silencing in these parasites but not for *VSG* ES silencing. We now show that DAC1 antagonizes basal telomeric silencing in bloodstream-form trypanosomes. In support of a specific role for DAC1 in this anti-silencing effect, we see that loss of this phenotype in insect-stage cells coincides with relocalization of DAC1 to the cytoplasm in this life-cycle stage. Interestingly, the class I HDACs, Rpd3 and HDAC1 play a similar telomeric anti-silencing role in yeast (Zhou *et al*., 2009) and in *Drosophila* (Doheny *et al*., 2008). Rpd3 has been proposed to mediate this effect by controlling the formation of transcription boundaries, which prevent Sir2-dependent silencing from spreading into euchromatic regions (Zhou *et al*., 2009). Thus, HAT1 and DAC1 may cooperate to regulate SIR2rp1-mediated silencing at *T. brucei* telomeres and this may reflect a role for histone acetylation in boundary element formation that is conserved from trypanosomes to metazoans. In this respect, it is also interesting that bloodstream-form cells defective in either HAT1 (Kawahara *et al*., 2008) or DAC1 (this report) also display nuclear DNA replication defects; a proportion of cells progress inappropriately through mitochondrial DNA division or mitosis in cells depleted for DAC1 or HAT1 respectively. These results suggest that the establishment of transcription boundaries is linked to DNA replication control in trypanosomes. Developmental control of DAC1 function indicates that the telomeric boundary is specific to the bloodstream form.

A small number of factors have now been linked to the process of telomeric *VSG* ES silencing and it was recently reported that nucleosomes are depleted at the active ES (Figueiredo and Cross, 2010; Stanne and Rudenko, 2010), but our understanding of the silencing mechanism remains incomplete. In this study, we demonstrate that DAC3 is required for *VSG* ES promoter silencing, but following DAC3 knockdown, transcription is attenuated through the polycistronic ES. This phenotype is observed in both life-cycle stages examined, consistent with the nuclear accumulation of DAC3 in both of these stages. Notably, a chromatin remodelling factor, TbISWI, has been linked to similar phenotypes (Hughes *et al*., 2007); depletion of TbISWI leads to derepression of a silent *VSG* ES promoter, but no derepression of *VSG*s at the distal end of the ES. This is consistent with the idea that ISWI remodelers organize nucleosomes at inactive regions lacking acetylation (Corona *et al*., 2002) but what factors are responsible for transcription attenuation within *VSG* ESs in the absence of TbISWI or DAC3? Our data indicate that neither SIR2rp1 nor DAC1 are required for this attenuation in DAC3-depleted cells. In fact, the only factors linked to transcription repression throughout the ES are repressor/activator protein 1 (Yang *et al*., 2009) and DOT1B (Figueiredo *et al*., 2008). Thus, transcription attenuation in cells depleted of DAC3 or TbISWI is likely to be dependent upon the action of these factors.

Histone deacetylases have emerged as potential new drug targets in protozoan parasites. Our study reveals essential HDACs in African trypanosomes that display distinct functions in antagonizing basal telomeric silencing and facilitating telomeric *VSG* ES silencing. These findings provide a mechanistic basis for the development of new parasite deacetylase inhibitors.

## Experimental procedures

### *T. brucei* growth and manipulation

All cells were derived from *T. brucei* Lister 427 bloodstream-form MITat1.2 (clone 221a). The bloodstream-form cells were grown in HMI-11, transformed with linear DNA constructs and differentiated to the insect stage in DTM as described (Alsford *et al*., 2005; 2007;). Drugs were added 6 h post transfection at the following concentrations: phleomycin (CayLa), 2 µg ml^−1^; hygromycin (Sigma), 2.5 µg ml^−1^; blasticidin (Invitrogen), 10 µg ml^−1^; G418 (MBI Fermentas), 2 µg ml^−1^. Induction was carried out using tetracycline (Sigma) at 1 µg ml^−1^. Cell counts were carried out using a haemocytometer. Cell cycle analysis and flow cytometry were carried out as described (Kawahara *et al*., 2008).

### Plasmid construction

Genes or gene fragments were amplified by PCR from genomic DNA clones using Fusion high fidelity DNA polymerase (NEB) and specific oligonucleotides. pMalc2x (NEB) and pTrcHis-C (Invitrogen) were used for DAC1 and DAC3 expression in *E. coli* respectively. pRPa^TAGx^, pNAT^GFPx^ and pRPa^iSL^ were used for expression of N-terminal fusion proteins and for hairpin RNAi respectively (Alsford and Horn, 2008). pi*GFP:NPT* was derived from pi*RFP:PAC* (Glover *et al*., 2007). Briefly, *PAC* was replaced by *NPT* using digestion with NotI and ClaI, and *RFP* was replaced by *GFP* using digestion with NotI and HindIII. *NPT-* and *BLA*-targeting constructs (Alsford *et al*., 2007) were used for SIR2rp1 disruption. Site-directed mutagenesis was carried out using the QuikChange kit according to the manufactures' instruction (Stratagene). Briefly, 35 ng of pTrcHis-DAC3 was used as a template and 2.5 U of PfuUltra high-fidelity DNA polymerase was used for amplification. PCR was run as follows: initially 95°C for 30 s; then 16 cycles of 95°C for 30 s, 55°C for 60 s, 68°C for 6 min; and final extension 5 min at 72°C. PCR product was digested with 10 U of DpnI to remove the template. The digested mixture was cleaned with StrataClean resin (Stratagene) and transfected into XL1-Blue *E. coli* by electroporation. pTrcHisDAC3^H316A^ mutant clones were confirmed by sequencing. All oligonucleotide sequences are available upon request.

### Expression in *E. coli* and purification

For ^MBP^DAC1 expression and purification, pMalc2x-DAC1 was transformed into the protease-deficient strain, ER2508 (NEB) and cells at an OD_600_ of 0.5 were induced with 0.3 mM IPTG for 4 h. Protein purification was carried out according to standard procedures (NEB). For ^HIS^DAC3 and ^HIS^DAC3^H316A^ mutant expression and purification, pTrcHis-DAC3 and pTrcHis-DAC3^H316A^ were transformed into the BL21 strain (Invitrogen) and cells at an OD_600_ of 0.6 were induced with 1 mM IPTG for 2 h. Protein purification was carried out according to standard procedures (Qiagen). Protein concentrations were determined using the BCA protein assay kit (Thermo scientific). Proteins were stored in elution buffer plus 1 mM DTT and 10% glycerol at −80°C.

### HDAC activity assay

DAC1 and DAC3 activity assays were carried out by HDAC Colorimetric Assay, Drug Discovery Kit according to the manufacturer's instructions (Biomol). Briefly, 20 µl assay buffer and 5 µl purified protein were added to a 96-well plate, and then 25 µl substrate was added and mixed thoroughly (final concentration is 0.5 mM). Following incubation at 37°C for 30 min, 50 µl developer solution was added and incubated for another 10 min prior to reading the OD at 405 nm.

### DNA and RNA analysis

DNA sequencing was performed using a Thermo Sequenase dye terminator Kit (Applied Biosystems), a thermal cycler and an ABI Prism 377 automated sequencer according to the manufacturer's instructions. PCR and Northern analysis were carried out according to standard protocols and signals on Northern blots were quantified using a Phosphorimager (Amersham).

### Protein analysis

For Western blots, whole cell lysates were separated by SDS-PAGE and electroblotted using standard protocols. Antibodies used included rabbit polyclonal α-NPT (1:2000), mouse α-cMyc 9E10 (1:2000), mouse monoclonal α-Xpress (1:5000), rabbit polyclonal α-GFP (1:4000) and rabbit polyclonal α-*VSG2* (1:20000). Western blot signals were detected using an ECL + Kit (Amersham) according to the manufacturer's instructions. Immunofluorescence microscopy was carried out as described (Alsford *et al*., 2007). Briefly, cells were fixed with 2% formaldehyde for at least 1 h and washed twice with PBS and once with 1% BSA (in H_2_O) before drying on slides for at least 3 h. Cells were then permeablized with 0.5% Triton X-100 for 10 or 20 min and blocked in 30% FBS for 5 min. Indirect detection of GFP and MYC epitopes was carried out using α-GFP (1:200) and α-cMyc 9E10 (1:500) using standard protocols. Samples were mounted in Vectashield (Vector Laboratories) containing the DNA counterstain, 4′,6-diamidino-2-phenylindole. Slides were analysed on a Nikon Eclipse E600 epifluorescence microscope. Phase and fluorescence images were captured using a Coolsnap FX (Photometrics) CCD camera and processed in Metamorph 5.0 (Universal Imaging) and Photoshop Elements 2.0 (Adobe).
