# Supplementary material for: Histone deacetylases play distinct roles in telomeric VSG expression site silencing in African trypanosomes
Source: Mol Microbiol. 2010 Jul 22;77(5):1237–45. doi: 10.1111/j.1365-2958.2010.07284.x (PMC2941730; doi:10.1111/j.1365-2958.2010.07284.x)
Supplement: Supplementary file 1 [file mmi0077-1237-SD1.pdf]

Histone deacetylases play distinct roles in telomeric *VSG* expression site silencing in African trypanosomes

Qiao-Ping Wang, Taemi Kawahara and David Horn

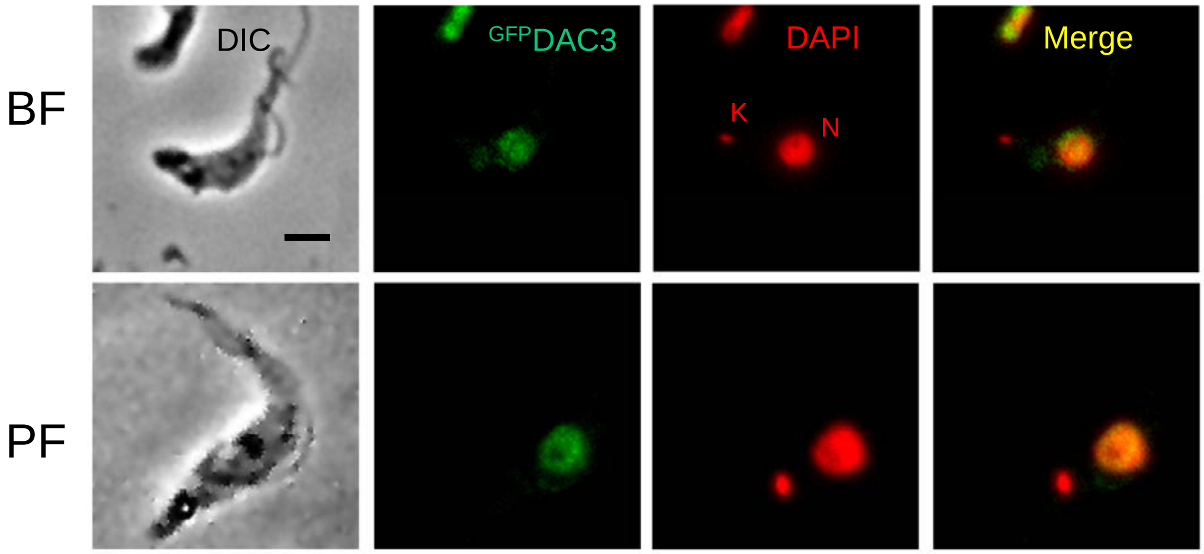

Figure S1: Localization of ^{GFP}DAC3 expressed from the native locus. Immunofluorescence detection was carried out using bloodstream form (BF) and procyclic form (PF) cells. N, nucleus; K, kinetoplast (mitochondrial DNA). Bar, 2µm.

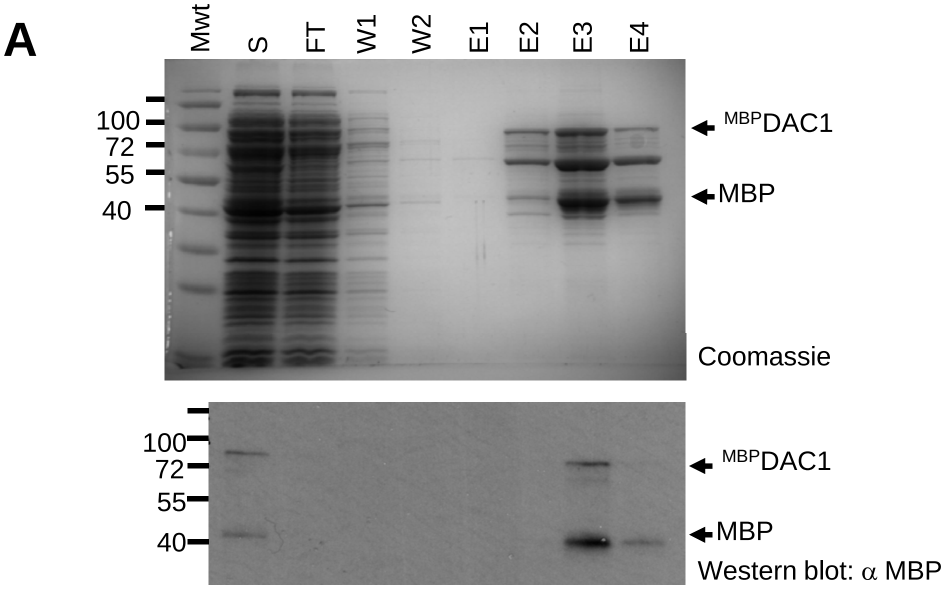

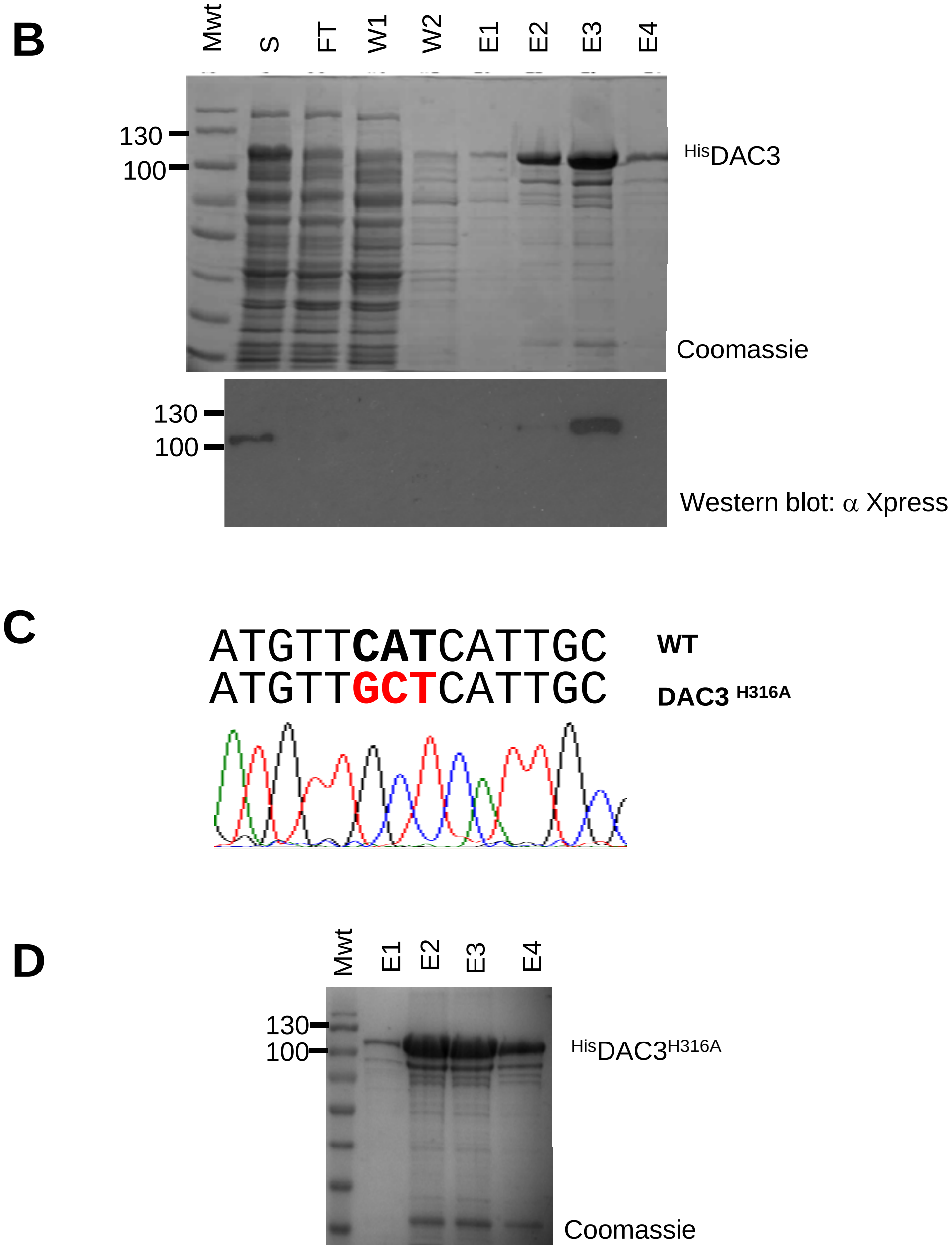

Figure S2: Expression and purification of DAC1 and DAC3 from *E. coli*. (A) ^{MBP}DAC1 was purified by affinity chromatography using amylose resin. Samples were separated on 10% SDS-PAGE gels. S, supernatent; FT, flow-through; W, wash; E, elution. Three major products eluted from the amylose resin. (B) ^{His}DAC3 was purified by affinity chromatography using Ni-NTA columns. Other details as in A. (C) The DAC3^{H316A} mutant was confirmed by sequencing; altered codon indicated in red. (D) The DAC3^{H316A} mutant was purified as in B.

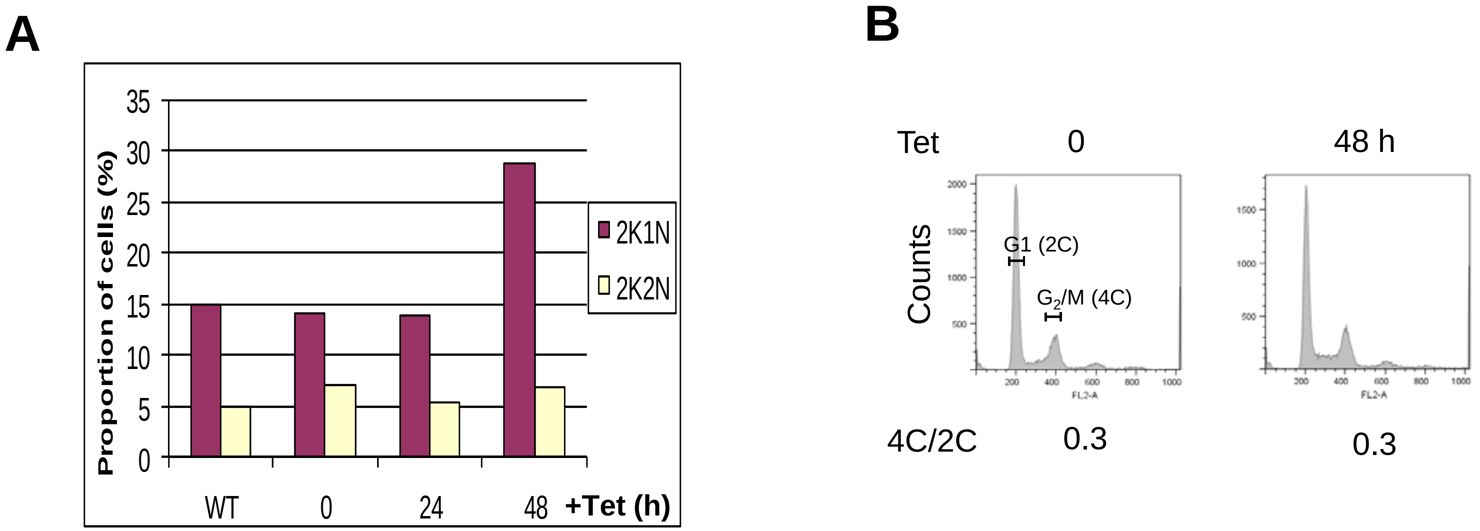

Figure S3: DAC1 appears to be required for efficient nuclear DNA replication in bloodstream form cells.
(A) Nuclear and mitochondrial (kinetoplast) DNA, stained with DAPI, were used as cytological markers to define position in the cell cycle. DAC1-knockdown in bloodstream form cells lead to an increase in the proportion of cells with a segregated mitochondrial genome. 2K1N indicates cells with two kinetoplasts and a single nucleus corresponding to nuclear G_2. n > 300 cells for each sample/time point.
(B) Analysis of cellular DNA content by flow cytometry revealed no increase in the proportion of cells that had replicated the nuclear genome (4C or 4x haploid nuclear DNA content) in this population. Since nuclear DNA replication normally precedes mitochondrial DNA segregation, these results link DAC1 knockdown to a DNA replication defect. The proportion of 4C relative to 2C is indicated. n=30,000 cells.
